# Salicylic Acid-Mediated Disturbance Increases Bacterial Diversity in the Phyllosphere but Is Overcome by a Dominant Core Community

**DOI:** 10.3389/fmicb.2022.809940

**Published:** 2022-02-24

**Authors:** Stacey A. Vincent, Andreas Ebertz, Pietro D. Spanu, Paul F. Devlin

**Affiliations:** ^1^Department of Biological Sciences, Royal Holloway, University of London, Egham, United Kingdom; ^2^Department of Life Sciences, Imperial College London, London, United Kingdom

**Keywords:** phyllosphere, salicylic acid, microbiome, plant, immune response, metabarcoding, diversity, bacteria

## Abstract

Plant microbiomes and immune responses have coevolved through history, and this applies just as much to the phyllosphere microbiome and defense phytohormone signaling. When in homeostasis, the phyllosphere microbiome confers benefits to its host. However, the phyllosphere is also dynamic and subject to stochastic events that can modulate community assembly. Investigations into the impact of defense phytohormone signaling on the microbiome have so far been limited to culture-dependent studies; or focused on the rhizosphere. In this study, the impact of the foliar phytohormone salicylic acid (SA) on the structure and composition of the phyllosphere microbiome was investigated. 16S rRNA amplicons were sequenced from aerial tissues of two *Arabidopsis* mutants that exhibit elevated SA signaling through different mechanisms. SA signaling was shown to increase community diversity and to result in the colonization of rare, satellite taxa in the phyllosphere. However, a stable core community remained in high abundance. Therefore, we propose that SA signaling acts as a source of intermediate disturbance in the phyllosphere. Predictive metagenomics revealed that the SA-mediated microbiome was enriched for antibiotic biosynthesis and the degradation of a diverse range of xenobiotics. Core taxa were predicted to be more motile, biofilm-forming and were enriched for traits associated with microbe-microbe communication; offering potential mechanistic explanation of their success despite SA-mediated phyllospheric disturbance.

## Introduction

Plants and animals have coevolved throughout history with functionally diverse colonizing microbes which modulate host fitness and environment-holobiont interactions (Limborg and Heeb, 2018). The microbiome of plants broadly comprises the below-ground rhizosphere and aerial phyllosphere, of which the rhizosphere is better characterized (Turner et al., 2013; Perazzolli et al., 2014). The phyllosphere microbiome is dynamic and variable due to environmental exposure and the low nutrient availability in aerial plant matter when compared to the nutrient-rich soil environment in the rhizosphere (Maignien et al., 2014; Bringel and Couée, 2015; Singh et al., 2019). Phyllosphere microbiota are exposed to fluctuations in wind speed, temperature, light, humidity, UV radiation and rainfall which can temporally alter the microbiome composition (Carvalho and Castillo, 2018; Dastogeer et al., 2020; Sivakumar et al., 2020). These conditions make the phyllosphere prone to stochastic colonization processes such as dispersal and ecological drift (Maignien et al., 2014). Despite the harsh, nutrient-poor environment in the phyllosphere, microbial community assembly is not considered to be an entirely random process; and is driven by other factors, including the host age, leaf physiology and genotype (Vorholt, 2012; Wagner et al., 2016; Singh et al., 2018; Chen T. et al., 2020; Shakir et al., 2020).

Plant phyllospheres are colonized by diverse microbial taxa, including nematodes, archaea, algae and protists; however, the most prevalent microorganisms, both with respect to abundance and current scientific understanding, are fungi and, particularly, bacteria (Vorholt, 2012; Trivedi et al., 2020). The stable phyllosphere confers fitness benefits to its host plant, including the acquisition and biological availability of essential elements; UV and drought tolerance; growth promotion; and the competitive exclusion of plant pathogens (Vorholt, 2012; Carvalho and Castillo, 2018; Santana et al., 2018; Stone et al., 2018). Interactions between the host plant and the phyllosphere microbiome maintain a homeostatic state within the microbial community and, as in many ecological communities, in the homeostatic phyllosphere there is often a prevalence of few, dominant microbial taxa (Vorholt, 2012; Kembel et al., 2014; Ottesen et al., 2016). However, microbial imbalance, known as “dysbiosis,” has been observed in the phyllosphere (Chen T. et al., 2020). This significant discovery, modulated by an underlying host genetic network, demonstrated that dysbiosis in the phyllosphere is correlated with lower relative abundance of members of the Phylum Firmicutes and reduced diversity, mirroring similar observations in clinical gut microbiome research (Rinninella et al., 2019; Chen T. et al., 2020). The transplantation of synthetic communities (SynComs) generated from a mutant with deficient immune signaling to a healthy plant line was additionally shown to negatively impact plant fitness (Chen T. et al., 2020). Microbial diversity in the environment has also been sometimes shown to be highest when disturbance is observed at intermediate intensity or frequency; and is associated with reduced stability and stochastic community assembly (Galand et al., 2016; Gibbons et al., 2016; Santillan et al., 2019). This phenomenon is known in wider ecology as the intermediate disturbance hypothesis (IDH), wherein disturbance is sufficient to result in niche diversification or partitioning, and the prevention of competitive exclusion by dominating taxa, resulting in the influx of opportunistic colonizers; but that also the disturbance is not severe or frequent enough to eliminate well-adapted species, thereby maximizing diversity (Connell, 1978; Bendix et al., 2017).

Disturbance in the form of phytohormone-mediated plant immunity has been shown to have complex regulatory effects on plant-microbe interactions (Kniskern et al., 2007; Carvalhais et al., 2015; Lebeis et al., 2015; Chen X. et al., 2020). The plant immune system has coevolved with the establishment of the plant microbiome (Turner et al., 2013) and is coordinated by the phytohormones salicylic acid (SA), jasmonates (JAs), and ethylene (ET) (Pieterse et al., 2012; Ma and Ma, 2016). These hormones crosstalk to induce a multitude of responses and developmental processes *in planta*, enabling the plant to adapt to a range of abiotic and biotic stressors, including the response to microbial pathogens (Devoto and Turner, 2003; Yang et al., 2019). Defense against biotrophic pathogens is coordinated by SA, whilst JA and ET are associated with defense against pathogens with a necrotrophic lifestyle (Devoto and Turner, 2003; Ma and Ma, 2016; Han, 2017). SA additionally is involved in regulating systemic acquired resistance (SAR), a process that results from the hypersensitive response (HR) following effector-triggered immunity (ETI), conferring broad resistance to disease (Muthamilarasan and Prasad, 2013; Balint-Kurti, 2019; Tripathi et al., 2019). However, elevated SA signaling also results in attenuated JA responses and a trade-off with plant growth (Devoto and Turner, 2003; Van der Does et al., 2013; Huot et al., 2014). Previous studies have shown that both SA and JA can modulate the plant microbiome and that plant immune signaling acts as a selective pressure in community assembly (Kniskern et al., 2007; Carvalhais et al., 2015; Lebeis et al., 2015; Chen X. et al., 2020). SA has been shown to be necessary for normal root endosphere formation (Lebeis et al., 2015). However, in *Arabidopsis thaliana (A. thaliana)* mutants that constitutively express SA, root endosphere bacterial community diversity was reduced and a shift in community composition was observed; with some taxa putatively dependent on SA signaling for colonization and endosphere microbiota formation, and others no longer colonizing the plant in the presence of elevated SA signaling (Lebeis et al., 2015). Whilst the role of SA in modulating rhizosphere community assembly and diversity has been reported (Lebeis et al., 2015), there is an absence of existing NGS-based, culture-independent research on the impact of SA on the total leaf phyllosphere, despite its status as a foliar phytohormone.

The *A. thaliana* double mutant *fhy3 far1* exhibits constitutive SA signaling through the loss-of-function of the FAR-RED ELONGATED HYPOCOTYL 3 (FHY3) and FAR-RED IMPAIRED RESPONSE 1 (FAR1) proteins that negatively regulate SA accumulation and are involved in far-red light responses via phyA signaling (Wang and Xing, 2002; Hudson et al., 2003; Lin et al., 2007; Siddiqui et al., 2016; Liu et al., 2019). *fhy3 far1* mutants also display reduced growth and leaf lesions characteristic of the HR, both of which are exacerbated in short day (SD) conditions as well as enhanced production of reactive oxygen species (ROS) (Ma et al., 2016; Wang et al., 2016). The gene LESION STIMULATING DISEASE 1 (LSD1) negatively regulates ROS-and SA-mediated cell death (Huang et al., 2010; Li et al., 2013). The corresponding *lsd1* mutant displays runaway cell death (RCD) and SA accumulation following stress exposure (Mateo et al., 2004; Huang et al., 2010; Li et al., 2013). The constitutive SA signaling mutant *cpr5*, which demonstrates phenotypic pleiotropies (Jing and Dijkwel, 2008), has been previously used to investigate the bacterial leaf and root endospheres (Lebeis et al., 2015; Pfeilmeier et al., 2021). Both the *fhy3 far1* and *lsd1* mutants demonstrate phenotypic pleiotropies that are divergent, with only mutations that affect SA signaling common to both. Therefore, any similar modulations in microbial composition and structure that occur are likely due to the impact of SA. However, previous research suggests that *fhy3 far1* demonstrates a greater fold-change of SA and ROS accumulation compared to its wild type (WT) than *lsd1* (Huang et al., 2010; Wang et al., 2016; Bernacki et al., 2019, 2021). Moreover, these two mutants originate from different ecotype backgrounds, thereby enabling the investigation of any interplay between genotype and SA signaling. The *fhy3 far1* and *lsd1 A. thaliana* mutants are, therefore, proposed as models to robustly elucidate the impact of SA signaling on the phyllosphere microbiome both by enhanced and accumulated SA signaling through the use of 16S rRNA amplicon sequencing.

## Materials and Methods

### Plant Growth Conditions

*A. thaliana* seeds were sown on damp soil without prior sterilization of either compost or seed. The compost used consisted of John Innes No.3 soil, Levington M3 soil and perlite (6:6:1 vol/vol, approximately). Plants grown in SD conditions were kept at 20°C at a Photon Flux Density (PFD) of 127 μmol m^–2^ s^–1^ and subjected to 8 h/16 h light/dark cycles and germinated in a Photon Systems Instrument^®^ FytoScope FS 130 cabinet. The proportional RGB composition of the LED intensities was adjusted to equal proportions of red (627 nm), green (530 nm), and blue light (470 nm) to mimic that of white light. Plants grown in LD conditions were germinated in a temperature-controlled growth room on a 16 h/8 h light/dark cycle at 20°C. Seeds used in this study had been harvested from parental lines grown in the same conditions across multiple generations to control for the initial inoculum. SD experiments were conducted in three independent sowing rounds. Three repeat experiments were carried out to form three completely independent biological replicates for each comparison.

### DNA Extraction

For each biological replicate, frozen aerial plant tissue from three individual plants of each genotype 35 days after sowing (DAS) was homogenized by adding glass silica beads at a sufficient quantity to fully cover the lysis buffer added downstream; flash-frozen again and vortexed using the Scientific Industries™ Vortex-Genie™ 2 at maximum rpm for 30 s. The samples were immediately flash-frozen a third time and the lysis buffer from the Qiagen^®^ DNeasy^®^ Plant Mini Kit was added. The samples were vortexed for a further 3.5 min, or until the tissue was completely homogenized. The remaining steps of the DNA extraction were carried out according to the manufacturer’s instructions.

### Polymerase Chain Reaction Amplification

Universal 16S rRNA primer pairs targeting conserved regions were used to amplify extracted DNA. Two combinations were used to prepare sequences for NGS metabarcoding: the first using the 27F (5′-AGA GTT TGA TCC TGG CTC AG-3′) and 783R (5′-CTA CCV GGG TAT CTA ATC CBG-3′) primer pair combinations targeting the V1-V4 regions for No-0 and *fhy3 far1 samples*; and the second using the 799F (5′-AAC MGG ATT AGA TAC CCK G-3′) and 1193R (5′-ACG TCA TCC CCA CCT TCC-3′) primer pair combinations, targeting the V5-V7 16S rRNA regions for Col-0 and *lsd1* samples. All polymerase chain reactions (PCRs) were carried out in triplicate 25 μl reactions.

A hemi-nested PCR was used for V1-V4 regions to amplify bacterial DNA in order to avoid isolation of mtDNA and cpDNA which have homology for conserved 16S regions. In detail, bacterial DNA was amplified using the 27F and 1492R (5′-GGT TAC CTT GTT ACG ACT T-3′) primer pair, which target V1-V9. The PCR was carried out in reactions each containing 1X GoTaq^®^ Hot Start Green Master Mix; 2.6 μM MgCl2; 2 μM DMSO; 0.1 μM 27F primer; 0.1 μM 1492R primer and 2 μl of DNA template. PCR cycling conditions were: an initial denaturation at 94°C for 4 min; touch-down annealing from 60 to 50°C, decreasing in 1°C increments per cycle for 1 min; and extending at 72°C for 1 min. After reaching the final annealing temperature of 50°C this was repeated for 20 cycles with a final extension step at 72°C for 7 min. Sequences were then separated on a 1% low melting temperature agarose gel; and the band corresponding to the expected amplicon size was excised to separate the desired sequences from mtDNA, which has a larger expected amplicon size with this primer pair. The DNA was then purified from the gel using the Qiagen^®^ Gel Extraction kit, according to the manufacturer’s instructions and used as a template for the second round of PCR. PCR conditions as previously stated were used, with the degenerate, chloroplast-excluding reverse primer 783R to target V1–V4. PCR products of were then purified using the Qiagen^®^ PCR Purification Kit according to the manufacturer’s instructions. For the 799 F/1193 R combination, the same PCR was used without the use of DMSO and MgCl_2_ additives and touch-down annealing from 63 to 53°C, repeating for 30 cycles at the final annealing temperature. Amplicons were extracted from a gel as previously described without a second PCR step.

### Illumina MiSeq Next-Generation Sequencing

16S rRNA amplicons for each sample were used as input for library preparation. DNA libraries were prepared from amplicons by tagmentation using the Nextera™ DNA Flex Library Prep Kit from Illumina according to the manufacturer’s instructions. Library concentrations were quantified using Qubit and the library length was assessed using the Agilent TapeStation, and subsequently sequenced on the Illumina MiSeq platform, generating paired-end reads. The quality of output reads was assessed using FastQC (v.0.72 for Galaxy) (Andrews, 2010; Afgan et al., 2018).

### Bioinformatics Analysis

Microbiome analysis was carried out with mothur on the command line (v.1.44.3) using modifications to the mothur standard operating procedure (Schloss et al., 2009) using default parameters, unless specified. Firstly, forward and reverse reads were aligned into contigs using the Needleman alignment method. Screening was performed to remove reads that contained ambiguous sequences, including those from poor quality, non-overlapping reads, and homopolymers longer than the reference database later used for classification. Chimeric sequences were identified using the VSEARCH tool (Rognes et al., 2016) and also removed. Bacterial 16S rRNA sequences were then classified to the lowest possible taxonomic level using the mothur-formatted SILVA SEED alignment and taxonomy reference files v.138 (Quast et al., 2013) using the Wang classification algorithm with an 80% bootstrap confidence cut-off. Undesired taxa, such as cpDNA and mtDNA were subsequently removed. To overcome clustering issues resulting from the different primer pairs used in this study, the remaining sequences were binned according to phylotype. A subsampling approach was implemented for subsequent analysis to avoid issues with uneven sample size and rare reads, in accordance with the mothur standard operating procedure (Schloss et al., 2009). Data subsampling was repeated three times and produced similar results (Supplementary Figure 2). Alpha and beta diversity analyses was additionally carried out in mothur using the Good’s coverage index (Good and Toulmin, 1956), Chao1 estimator of species richness (Chao, 1984), Shannon evenness and Simpson diversity indexes (Shannon, 1948; Simpson, 1949) for alpha diversity; and the Bray-Curtis dissimilarity index (Bray and Curtis, 1957) for beta diversity. For comparisons of Simpson diversity indices, replicates were averaged for each genotype and compared using the Past4 Diversity *t*-test function (Hutcheson, 1970). Statistical comparisons of relative abundances were carried out using the *Metastats* algorithm in mothur (Schloss et al., 2009; White et al., 2009).

### Predictive Metagenomics

Predicted metagenomes were obtained from representative phylotype sequences and corresponding abundance tables using the Piphillin algorithm (Iwai et al., 2016; Narayan et al., 2020) at a 97% identity cutoff and cross-referenced with the KEGG database (May 2020 release) (Kanehisa and Goto, 2000; Kanehisa, 2019; Kanehisa et al., 2021). Statistical comparisons of the output count tables for both KEGG orthologs and pathways was carried out using DESeq2 using the poscount size factor estimator (Love et al., 2014). Reported *p*-values were values adjusted using the Benjamini-Hochberg false discovery rate (FDR) correction (Benjamini and Hochberg, 1995).

## Results

In order to assess the impact of SA and its potential role in mediating disturbance on the phyllosphere microbiome, NGS metabarcoding was performed on amplicons of bacterial 16S rRNA sequences extracted from aerial tissue 35 DAS on two *A. thaliana* mutants that exhibit elevated SA signaling, *fhy3 far1* and *lsd1*. Comparisons between the microbiomes of three biological replicates of the *fhy3 far1* and *lsd1* mutant lines and their WT background ecotypes (No-0 and Col-0, respectively) were performed using a phylotype-based approach due to the different 16S rRNA regions used for each pair. Comparisons between *fhy3 far1* and No-0 were performed across three independent experiments. A total of 286,931 high-quality, classified 16S rRNA sequences were obtained across the 12 samples (Table 1). Good’s coverage values (Good and Toulmin, 1956) were above 0.98 across all samples.

**TABLE 1:** Number of high-quality, classified 16S rRNA sequences and Good’s coverage produced by NGS per sample.

Ecotype background	Sample	No. of 16S sequences	16S Good’s coverage
No-0	No-0 replicate 1	11,319	0.993
	No-0 replicate 2	4,988	0.995
	No-0 replicate 3	13,676	0.997
	*fhy3 far1* replicate 1	57,858	0.988
	*fhy3 far1* replicate 2	5,880	0.991
	*fhy3 far1* replicate 3	6,162	0.994
Col-0	Col-0 replicate 1	33,567	0.994
	Col-0 replicate 2	62,974	0.994
	Col-0 replicate 3	4,583	0.989
	*lsd1* replicate 1	15,370	0.988
	*lsd1* replicate 2	12,946	0.991
	*lsd1* replicate 3	57,608	0.991
Total		286,931	

### Salicylic Acid Signaling Increases Diversity in the Phyllospheric Bacterial Community

**FIGURE 1:**
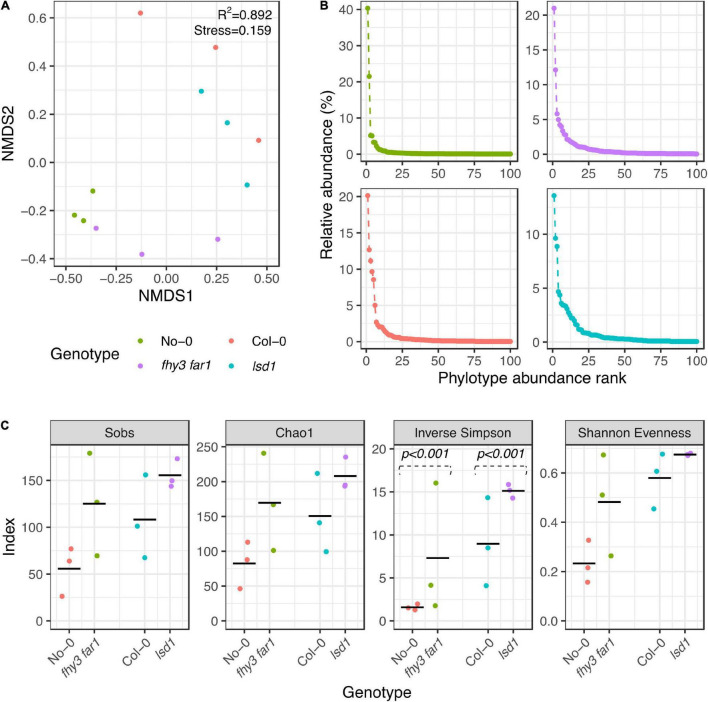
Alpha and beta diversities of phyllospheric bacterial communities in wild type and salicylic acid signaling mutants. **(A)** Non-metric multidimensional scaling of Bray-Curtis dissimilarities (Bray and Curtis, 1957) of phyllospheric bacterial communities across all four genotypes. Analysis carried out using 10 iterations with ε = 1e-12. **(B)** Ranked abundances of 100 most abundant phylotypes. **(C)** Alpha diversity estimations of bacterial phyllospheric communities. The Sobs calculator shows the number of phylotypes identified; the Chao1 estimator shows the estimated species richness (Chao, 1984); and the inverse Simpson and Shannon evenness index show the community diversity and richness, respectively (Shannon, 1948; Simpson, 1949). Statistical comparison of Simpson diversity index values was carried using the Past4 Diversity *t-*test function (Hutcheson, 1970) on averaged phylotype counts for each genotype. All other analyses and initial index values were calculated using mothur (v.1.44.3) (Schloss et al., 2009) from bacterial 16S rRNA sequences present in the phyllosphere microbiomes of four *A. thaliana* genotypes (*n* = 3 for all genotypes) 35 DAS. Samples were subsampled down to the size of the sample with the lowest number of sequences before calculating.

In order to assess the impact of constitutive or accumulative SA signaling on the phyllosphere microbiome community structure, alpha and beta diversity analysis was carried out. Analyses were performed using subsampled data to account for uneven read numbers across samples. Ordination of beta diversities was implemented to assess dissimilarity coefficients between the phyllosphere microbiomes of the mutant and WT genotypes using non-metric multidimensional scaling (NMDS) of the Bray-Curtis dissimilarity statistic (Bray and Curtis, 1957; Figure 1A). The stress values generated by the algorithm for 16S rRNA sequences (lowest stress = 0.159) suggested that a fair degree of the variance was explained by the model. In general, replicates from the same genotype clustered together. The ordination analysis additionally showed that samples from the same background ecotype clustered separately from those in the other background, thereby demonstrating differences in the phyllosphere microbiome both as a consequence of ecotype and mutation. Analysis of ranked abundance curves across all four genotypes revealed that the most abundant phylotypes in the WT were potentially more dominant than in the SA mutant lines (Figure 1B), constituting larger percentages of the overall relative abundance. More intermediately-abundant taxa were observed in both *fhy3 far1* and *lsd1* than in either WT genotype. Alpha diversity analysis additionally revealed SA mutation-induced changes to community richness, evenness and diversity (Figure 1C). Species richness was determined using the number of observed phylotypes at the genus level and the Chao1 estimator for species richness (Chao, 1984); and community diversity and evenness measured using the inverse Simpson’s diversity index (SDI) and Shannon’s evenness index (SEI), respectively (Shannon, 1948; Simpson, 1949). The bacterial communities of *fhy3 far1* and *lsd1* showed an increase in estimated richness, evenness and diversity compared to their WT counterparts. This shift in diversity remained consistent when data subsampling was repeated (Supplementary Figure 2).

### Host Genotype and Salicylic Acid Signaling Drive Phyllosphere Community Assembly

**FIGURE 2:**
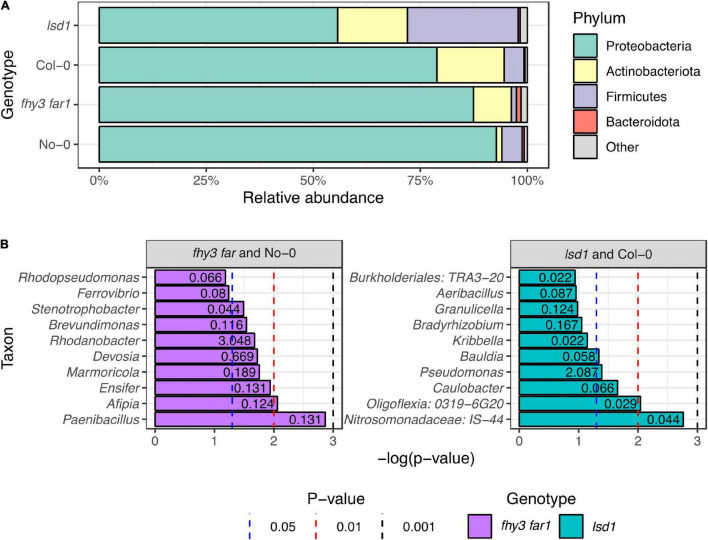
Phylotype abundances in phyllospheric bacterial communities in wild type and salicylic acid signaling mutants. **(A)** Relative abundances of classified bacterial phyla across all four genotypes. Classification of 16S rRNA sequences was carried out using the SILVA database (v.138) in mothur (v.1.44.3) (Schloss et al., 2009; Quast et al., 2013). **(B)** Taxa identified as differentially abundant in *fhy3 far1* and *lsd1* samples compared to their WT background ecotypes (No-0 and Col-0, respectively). Numbers presented on barcharts represent the relative abundance of the taxon (%). Statistical analysis carried out using the *Metastats* algorithm in mothur (v.1.44.3) (Schloss et al., 2009; White et al., 2009) on subsampled data. Sequences were isolated from aerial tissue 35 DAS (*n* = 3 for all genotypes).

Phyllosphere microbiome sequences were classified to the phylum level in No-0, *fhy3 far1*, Col-0 and *lsd1* and examined to assess the distribution of the relative abundances of the most prevalent phyla (Figure 2A). Each of the analyzed genotypes were dominated by bacteria belonging to the Phylum Proteobacteria. Both mutant lines exhibited a reduction in the relative abundance of Proteobacteria in the phyllosphere with respect to their WT counterparts, which was particularly evident between the Col-0 (79%) and *lsd1* (56%) samples. The Actinobacteriota, Firmicutes and Bacteroidota were the second, third and fourth most relatively abundant phyla overall, respectively. The Actinobacteria were more abundant in *fhy3 far1* (9%) with respect to No-0 (1%), whereas there was little difference in their relative abundance between *lsd1* and Col-0. *lsd1* had the highest relative abundance of Firmicutes (26%); however, *fhy3 far1* in fact had fewer phyllospheric Firmicutes than its WT line (1 and 5%, respectively). The Bacteroidota were most abundant in *fhy3 far1*, constituting just over one percent of the community, but constituting less than one percent of the relative abundance of bacteria in all other lines. Both mutant lines had a greater abundance of phyla that were less abundant across the samples (1% in *fhy3 far1* and 2% in *lsd1*; grouped as “other” in Figure 2A) than the two WT genotypes (< 1% in both). To assess the taxa that are differentially abundant in *fhy3 far1* and *lsd1* with respect to their WT counterparts, the *Metastats* algorithm (White et al., 2009) was implemented, which uses Fisher’s exact test to calculate differences in count data and is optimized for low-abundance features. The 10 differentially abundant taxa with the lowest *p*-values that could be classified to the genus level are reported (Figure 2B). This revealed that none of the significant genera were more abundant in WT genotypes than in the two mutant lines. With the exception of *Rhodanobacter* in *fhy3 far1* and *Pseudomonas* in *lsd1*, the significant genera were all rarely abundant (< 1% of total relative abundance), each constituting less than one percent of the mean relative abundances across the three biological replicates for that genotype. The most significantly differentially abundant genera in the *fhy3 far1* phyllosphere microbiome were *Paenibacillus, Afipia* and *Ensifer*; whereas, for *lsd1* these were IS-44 (of the Nitrosomonadaceae family), 0319-6G20 (of the Oligoflexia class) and the *Caulobacter* genus.

### Salicylic Acid Signaling Results in Colonization by Rare Taxa and the Retention of a Dominant Core Community in the Phyllosphere

**FIGURE 3:**
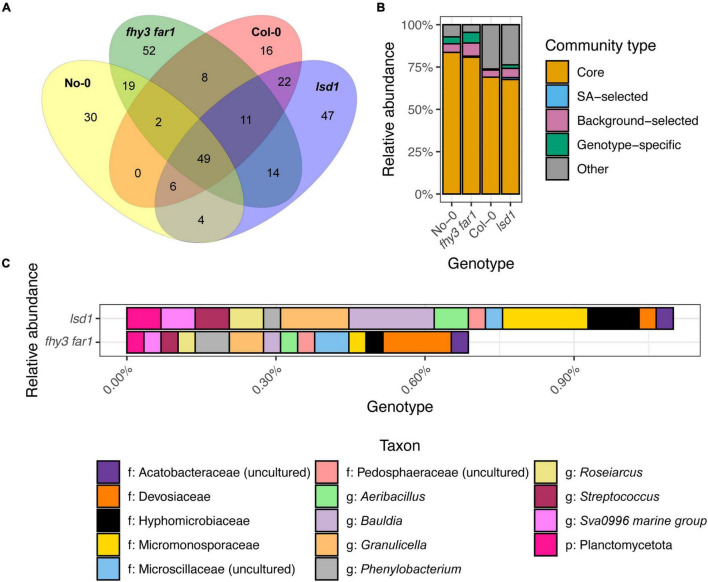
Distribution of phyllospheric bacterial communities across wild type and salicylic acid signaling mutants. **(A)** Venn diagram showing the overlap of phylotypes across the two WT ecotypes and two SA signaling mutants, including the 49 “core” phylotypes present in all four genotypes; 14 “SA-selected” phylotypes present only in the SA signaling mutant phyllospheres; and unique phylotypes in each genotype phyllosphere. Phylotypes were identified using mothur (v.1.44.3) (Schloss et al., 2009) from bacterial 16S rRNA sequences present in the phyllosphere microbiomes of four *A. thaliana* genotypes 35 DAS. Samples were subsampled down to the size of the sample with the lowest number of sequences before plotting. Phylotypes classified to at least the phylum level were selected for consideration. **(B)** Relative abundances of the core, SA-selected and unique communities across all four genotypes. **(C)** Relative abundances of the 14 SA-selected phylotypes in *lsd1* and *fhy3 far1*.

**FIGURE 4:**
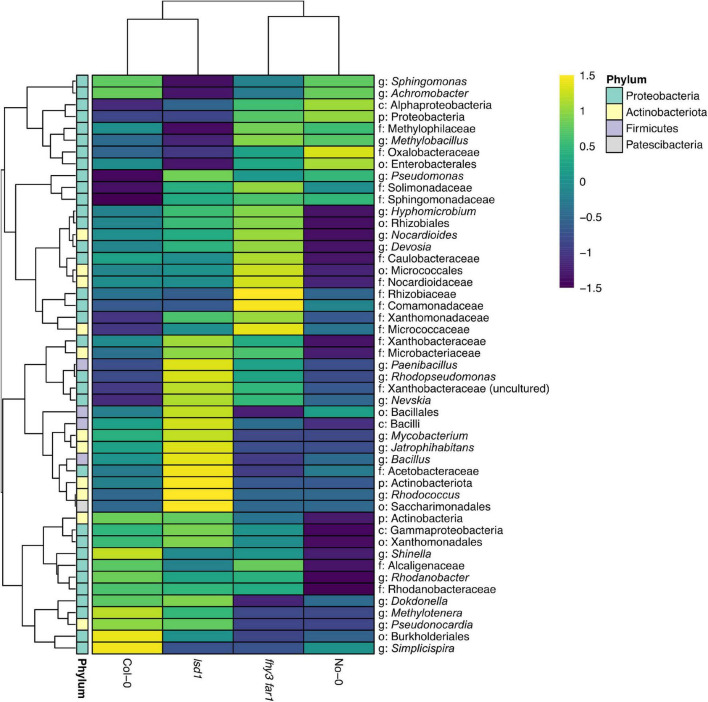
Hierarchical clustering of the relative abundance of core phyllospheric phylotypes. Euclidean clustering of the log^10^ relative abundances of the 49 core bacterial phyllospheric phylotypes, classified to at least the phylum level, present in both WT ecotypes and both SA signaling mutants. Phylotypes were classified using mothur (v.1.44.3) (Schloss et al., 2009) and the SILVA database (v.138) (Quast et al., 2013) from bacterial 16S rRNA sequences present in the phyllosphere microbiomes of four *A. thaliana* genotypes 35 DAS.

Comparisons of the presence and absence of phylotypes across genotypes revealed a co-factorial impact on phyllosphere microbiome assembly, but with a retained core community (Figure 3A). Following subsampling, 49 of the 286 total phylotypes observed were present across all genotypes. This remained consistent when subsampling was repeated (Supplementary Figure 2). Large overlaps were also apparent between WT and SA mutants of the same ecotype background that were not present in the other background (19 and 22 for No-0 and Col-0, respectively). *fhy3 far1* and *lsd1* had more unique phylotypes in their phyllospheric bacterial communities than their respective WTs; and were additionally observed to share 14 phylotypes that were not present in either WT that are putatively selected for, directly or indirectly, by SA signaling. No phylotypes were observed in both No-0 and Col-0 but absent in both *fhy3 far1* and *lsd1*. Further examination of the relative abundances of the 49 core, 14 SA-selected and 19 or 22 ecotype background-selected community types revealed that the SA mutants exhibited a small reduction in the relative abundance of core phylotypes, but the core microbiota still dominate the phyllosphere regardless of SA signaling (Figure 3B). A similar effect was observed when only comparing within the same ecotype background (Supplementary Figure 3). Both SA mutants exhibited greater relative abundance of phyllospheric bacteria shared only in the same ecotype background, as well as phylotypes unique to that genotype. The 14 SA-selected phylotypes present in both *lsd1* and *fhy3 far1* were observed to be rare in abundance. Further examination revealed that these included the *Aeribacillus, Bauldia, Granulicella, Phenylobacterium, Roseiaracus, Streptococcus*, and Sva0996 marine group (of the Acidimicrobiales order) genera (Figure 3C). Variation in the relative abundances of the core community types was seen to be impacted by ecotype, with the Col-0 genotypes showing reduced abundance of the core microbiota in comparison to the No-0 genotypes. Hierarchical clustering of the core taxa additionally demonstrated that ecotype, rather than elevated SA signaling, had a larger impact on the relative abundances of core phylotypes, with genotypes from the same ecotype background clustering together (Figure 4).

### The Phyllosphere Core Community Differs in Function From Salicylic Acid-Selected Microbes

**FIGURE 5:**
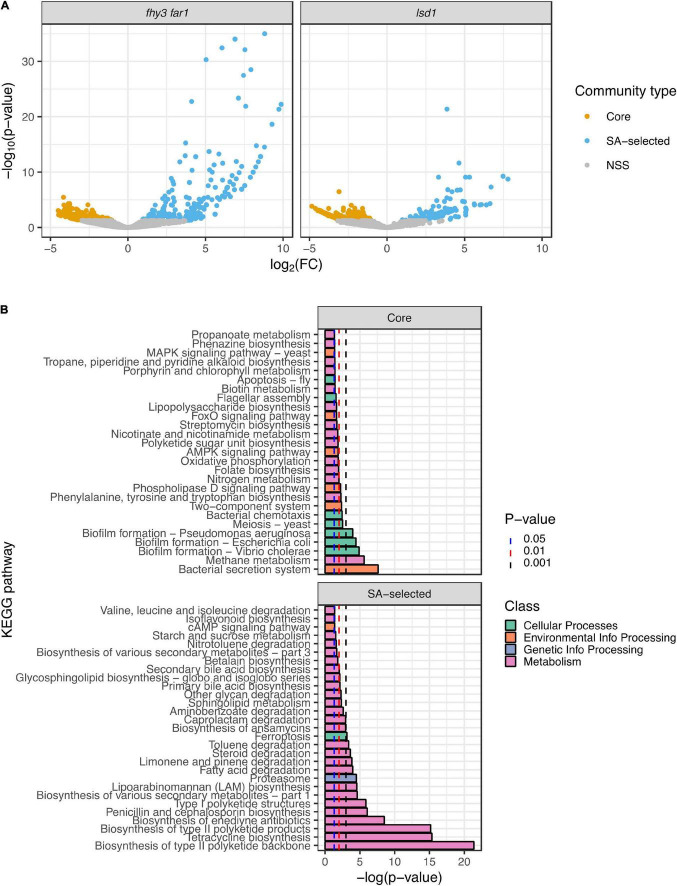
Predictive metagenomic analysis of functional traits of the core and salicylic acid-selected phyllospheric communities. **(A)** Volcano plots of differentially enriched KEGG orthologs predicted in the SA-selected and core phyllospheric bacterial communities in *lsd1* (*n* = 3) and *fhy3 far1* (*n* = 2). **(B)** Differentially enriched KEGG pathways predicted in the SA-selected (*n* = 5) and core (*n* = 8) phyllospheric communities. Functional prediction was carried out using the Piphillin algorithm (Iwai et al., 2016; Narayan et al., 2020) and mapped to the KEGG database (May 2020 release) (Kanehisa and Goto, 2000; Kanehisa, 2019; Kanehisa et al., 2021). Statistical comparisons were performed using DESeq2 (Love et al., 2014). Reported *p*-values were values adjusted using the Benjamini-Hochberg false discovery rate (FDR) correction (Benjamini and Hochberg, 1995).

In order to determine any functional differences between the core phyllospheric microbiota found across all genotypes and those present only in both SA mutants, a predictive metagenomics approach was employed. Representative sequences from the 14 SA-selected phylotypes present in *lsd1* and *fhy3 far1*, as well as the 49 core phylotypes across all four genotypes, were mapped to KEGG orthologs and pathways using Piphillin (Iwai et al., 2016; Narayan et al., 2020). Normalized core and SA-selected community KEGG ortholog and pathway counts were statistically compared using DESeq2 (Love et al., 2014). More highly statistically significant, differentially abundant predicted KEGG orthologs were observed in the SA-selected community than the core community in both *fhy3 far1* and *lsd1* (Figure 5A). Comparisons of the functional KEGG pathways of the core communities between each mutant and its WT revealed no significant differences in function (Supplementary Data). However, pathway comparison between SA-selected communities from both mutants and core communities from all four genotypes revealed differences in predicted functions in motility, antibiotic biosynthesis and metabolism (Figure 5B). The core community exhibited predicted functions in nicotinate, chlorophyll, nitrogen and methane metabolism; whereas the SA-selected community had predicted functions in glycan and lipid metabolism, as well as more enriched secondary metabolism. A number of pathways were also enriched in the core community concerned with cellular processes, including apoptosis, chemotaxis, flagellar assembly and biofilm formation in multiple species; but the only enriched cellular process in the SA-selected community was ferroptosis. Pathways involved in the antibiotic production were also predicted in the SA-selected community, including the biosynthesis of enediyne, tetracycline, ansamycin, penicillin and cephalosporin antibiotics pathways. The SA-selected community also had predicted enriched pathways involved in the degradation of various xenobiotic compounds, including aminobenzoate, steroids, toluene and caprolactam.

## Discussion

SA signaling is an important, intrinsic response *in planta* that regulates plant defenses and the establishment of SAR to modulate plant-pathogen interaction (Muthamilarasan and Prasad, 2013; Balint-Kurti, 2019; Tripathi et al., 2019). Culture-dependent studies carried out on the phyllosphere have shown that SA signaling modulates leaf endophytic communities (Kniskern et al., 2007). Historically, culture-independent studies of the impact of SA have been largely limited to the rhizosphere or root endosphere, despite the status of SA as a foliar phytohormone (Lebeis et al., 2015) and the emerging understanding of the importance of the phyllosphere as an antagonist for pathogens and its potential for dysbiosis (Vorholt, 2012; Carvalho and Castillo, 2018; Santana et al., 2018; Stone et al., 2018; Chen T. et al., 2020). The potential for phytohormone signaling and plant immunity to coordinate homeostasis and dysbiosis in the plant phyllosphere has also been an area of considerable interest (Chen T. et al., 2020; Gupta et al., 2021; Pfeilmeier et al., 2021). A recent analysis which focused on the role of the plant NADPH oxidase, RBOHD, in maintaining the leaf endosphere additionally observed changes in the composition of communities at the phylum level in an elevated SA signaling mutant, but not in SA signaling-deficient mutants; suggesting that loss of basal SA signaling does not modulate the leaf bacterial community, whilst elevated SA does (Pfeilmeier et al., 2021). Here, we have expanded this to include a comprehensive analysis of the impact of elevated SA signaling on total phyllospheric community structure and function as well as a more detailed analysis of the effect of SA-driven changes to community composition by demonstrating common mutation-driven shifts in two ecotype backgrounds. Understanding the impact of SA-mediated defense signaling on the total phyllosphere microbiome provides an insight into the magnitude of this disturbance to the bacterial community; as well as how much of this has potentially been mitigated by the coevolution of plant immune signaling and plant microbiome establishment. Identification of traits that promote stable plant-microbe interactions in the presence of plant immune responses may also provide insight into potential mechanisms that can be exploited for microbiome engineering, as well as candidate taxa for novel biocontrol applications.

In this study, 16S rRNA profiling was implemented to assess the impact of SA signaling in two *Arabidopsis* ecotypes on phyllospheric bacterial community structure and composition; as well as the predicted function of both the stable core community and putatively opportunistic colonizers. The findings presented here identified that both the constitutive and accumulative SA response increased species richness and diversity; and resulted in the colonization of the phyllosphere by rare taxa. Quantification of total SA levels in these mutants in previous research suggests that *fhy3 far1* displays a larger fold change in SA accumulation compared to WT plants than *lsd1*, suggesting that this effect on diversity is not concentration-dependent (Huang et al., 2010; Wang et al., 2016; Bernacki et al., 2019). High bacterial community diversity is typically associated with increased stability, whilst disturbance results in lower diversity in soil microbiomes (Wagg et al., 2018). In the root endosphere of another pleiotropic constitutive SA-signaling mutant (*cpr5)*, reduced bacterial diversity was observed (Lebeis et al., 2015). Therefore, the increased diversity in the phyllosphere suggests a differential effect of SA on bacterial communities in roots and aerial tissue. Moreover, in the lettuce phyllosphere, increased diversity of transplanted communities did not confer greater stability to an inoculated microbe (Williams and Marco, 2014). It is considered that, as a form of stress the phyllosphere will have co-evolved with, SA signaling may be acting as a source of intermediate disturbance to the phyllosphere microbiome, maximizing community diversity.

Previous research has highlighted that early phyllosphere microbiome assembly is shaped by stochastic events as well as host genotype and resulting phenotype (Maignien et al., 2014; Li et al., 2018; Morella et al., 2020), and that there is variability in the late-term success of opportunistic colonizers to invade an established core community and limited influence of the host genotype on community assembly in later developmental stages (Carlström et al., 2019; Morella et al., 2020). Moreover, stress acclimation-mediated microbiome shifts have been previously shown to be ecotype-dependent (Etemadi et al., 2018). In this study, both SA mutants were seen to be colonized by rare taxa present in both mutants, as well as additional unique taxa that were not present in any other genotype. This could potentially suggest stochasticity in the SA-modulated phyllosphere microbiome, with SA signaling resulting in unstable phyllosphere colonization. However, our findings did also show that there was a highly abundant core community which was able to both overcome SA signaling responses, and which was additionally present in both ecotypes. These taxa may, therefore, be potential candidates to promote stable plant-microbe interactions in the phyllosphere during the establishment of SAR or SA-mediated stress signaling. The relative abundance of the core community, however, did appear to be shaped more by ecotype than SA signaling, which may have implications for cultivar selection to mitigate impact of SA-mediated stress signaling on the phyllosphere microbiome. Moreover, the lack of taxa common to both wild ecotypes that were absent in both mutants suggests there may be commonalities between the underlying mechanism that modulates resilience to SA-mediated disturbance and ecotype-dependent variation.

An investigation into the predicted function of both the core and SA-selected communities was carried out to determine whether any detrimental functions were gained from the additional taxa that colonized both SA signaling mutants; as well as to investigate whether any potentially beneficial traits were lost. Lack of significant differences in the core phyllosphere communities of both *lsd1* and *fhy3 far1* when compared to their respective WTs suggests that these core functions are not diminished. However, multiple significant differences in the predicted functions of the core and SA-selected communities suggests that SA signaling results in many gained functions, whether beneficial or detrimental to the host plant. Previous research has demonstrated that the bacterial phyllosphere microbiome has the capacity to both produce and metabolize antibiotics, but that the magnitude is dependent on extrinsic cues, including biotic stress responses (Chen et al., 2018; Qi et al., 2021; Yan et al., 2021). The research presented here predicts that SA signaling selects for bacteria associated with the biosynthesis of antibiotics. The SA-selected community additionally was predicted to have enriched secondary metabolism, which is indicative of bacteria in the stationary phase of growth; and is a means for microbes to compete and communicate with other microbes (Joyce et al., 2011; Sharrar et al., 2020). Enriched ferroptosis may also be indicative of the enhanced ROS production observed in *fhy3 far1* and *lsd1* and which has also been shown to be driving by the immune response *in planta* (Ma et al., 2016; Wang et al., 2016; Herlihy et al., 2020; Bernacki et al., 2021). These findings, coupled with the diverse xenobiotics predicted to be degraded in the SA-selected community, highlight mechanisms through which bacteria may be able to compete in the disturbed microbiome and the role of SA in plant-microbe-microbe interactions.

The core community was additionally predicted to be enriched in functions that may add to the current understanding of how microbes colonize the phyllosphere, and features which may confer resilience to SA signaling and ecotype variation. Notably, the core community was shown to have enriched functions in motility, communication and adherence. The aerial environment is harsh and dynamic (Singh et al., 2019), and the ability to form biofilms, including temporary biofilm establishment, has been previously shown to assist in phyllosphere colonization, especially amongst epiphytes (Morris et al., 1998; Fink et al., 2012; Chaudhry et al., 2021). The findings presented here suggest that biofilm formation in multiple species may act as a mechanism for overcoming SA-mediated disturbance in the phyllosphere. Chemotaxis and motility via flagellar assembly coordinated by two-component systems enable bacterial movement to more favorable leaf sites and nutrition sources, consequently, successful colonization and survival (Gao and Stock, 2009; Copeland et al., 2015; Thapa and Prasanna, 2018; Chaudhry et al., 2021).

The study outlined here demonstrates that the defense phytohormone SA acts as a source of disturbance to the bacteria in the phyllosphere and increases community diversity. In addition, elevated SA results in the colonization of the phyllosphere by rare, satellite taxa; but that, in addition, a highly abundant stable core community can overcome SA-mediated disturbance. Rare SA-selected colonizers and core taxa were also predicted to have different functional attributes, with the core community predicted to be motile, chemotaxing and biofilm-forming. The enrichment of these predicted functions in both the disturbed and non-disturbed microbiome across different ecotypes suggests that these features may confer stability and resilience to ecotype variation and plant immune responses. Moreover, these findings also provide insight into features that could have been selected for during the coevolution of plant immune responses and microbiomes to promote stability in the event of phytohormone-mediated defense responses.

Although similarities in the *fhy3 far1* and *lsd1* microbiomes are reported in these findings, both of the mutants exhibit altered SA through signaling through different mechanisms and thereby show different quantities of SA (Wang et al., 2016; Bernacki et al., 2019, 2021). Moreover, the effect of exogenous SA application on the phyllosphere has not yet been assessed using a culture-independent approach. Further investigation into SA-mediated dose-dependent changes in the phyllosphere microbiome would elucidate the hyperimmune vs. a low-level response. Moreover, as SA cross-talks with other phytohormones and acts in antagonism with JA to mediate biotroph and necrotroph defenses (Devoto and Turner, 2003; Ma and Ma, 2016; Han, 2017), the role of the resulting diminished JA response in regulating would further compliment the findings presented here. Previous research has highlighted the growth-defense tradeoff in *fhy3 far1* plants which show reduced growth and increased resistance to the bacterial pathogen *Pseudomonas syringae* pv. *tomato* (*P.s.t.*) DC3000 (Wang et al., 2016). Reduced growth in the host plants is associated with dysbiosis which has been reported in the phyllosphere (Chen T. et al., 2020; Pfeilmeier et al., 2021). The discovery of phyllospheric dysbiosis is a recently discovered phenomenon and, consequently, defined characteristics of dysbiosis are still emerging. *fhy3 far1* displays some of the traits associated with phyllospheric dysbiosis such as chlorosis, reduced abundance or Firmicutes and a smaller phenotype. This research has demonstrated that the abundance of Proteobacteria in both the *fhy3 far1* and *lsd1* microbiomes is also reduced, which is not characteristic of dysbiosis (Chen T. et al., 2020). It is not yet known how much the *fhy3 far1* microbiome contributes to either its smaller phenotype or enhanced disease resistance. The application of SA-selected SynComs or functional traits predicted in this study would be an area of interest for future consideration to elucidate this.

## Data Availability Statement

The data presented in the study are deposited in the NCBI SRA repository, accession number SUB10801179.

## Author Contributions

SV contributed to experimental design, carried out the experiments, and wrote the bulk of the manuscript. AE contributed to experimental design. PS contributed to supervision of the project. PD conceived the original idea and supervised the project. All authors discussed the results and contributed to the final manuscript.

## Conflict of Interest

The authors declare that the research was conducted in the absence of any commercial or financial relationships that could be construed as a potential conflict of interest.

## Publisher’s Note

All claims expressed in this article are solely those of the authors and do not necessarily represent those of their affiliated organizations, or those of the publisher, the editors and the reviewers. Any product that may be evaluated in this article, or claim that may be made by its manufacturer, is not guaranteed or endorsed by the publisher.
