# Supplementary material for: Salicylic Acid-Mediated Disturbance Increases Bacterial Diversity in the Phyllosphere but Is Overcome by a Dominant Core Community
Source: Front Microbiol. 2022 Feb 24;13:809940. doi: 10.3389/fmicb.2022.809940 (PMC8908428; doi:10.3389/fmicb.2022.809940)
Supplement: Supplementary file 1 [file Data_Sheet_1.docx]

**Salicylic acid-mediated disturbance increases bacterial diversity in the phyllosphere and is mitigated by a stable core community**

**Supplementary figures**

Fig. S1. Rarefaction analysis of phyllospheric 16S rRNA sequences extracted from two WT (No-0 and Col-0) and two mutant (*fhy3 far1* and *lsd1*) *A. thaliana* lines. Microbial DNA was isolated from aerial tissue 35 DAS in three biological replicates and subjected to NGS. Analysis carried out using mothur (v.1.44.3) (Schloss et al., 2009).

**
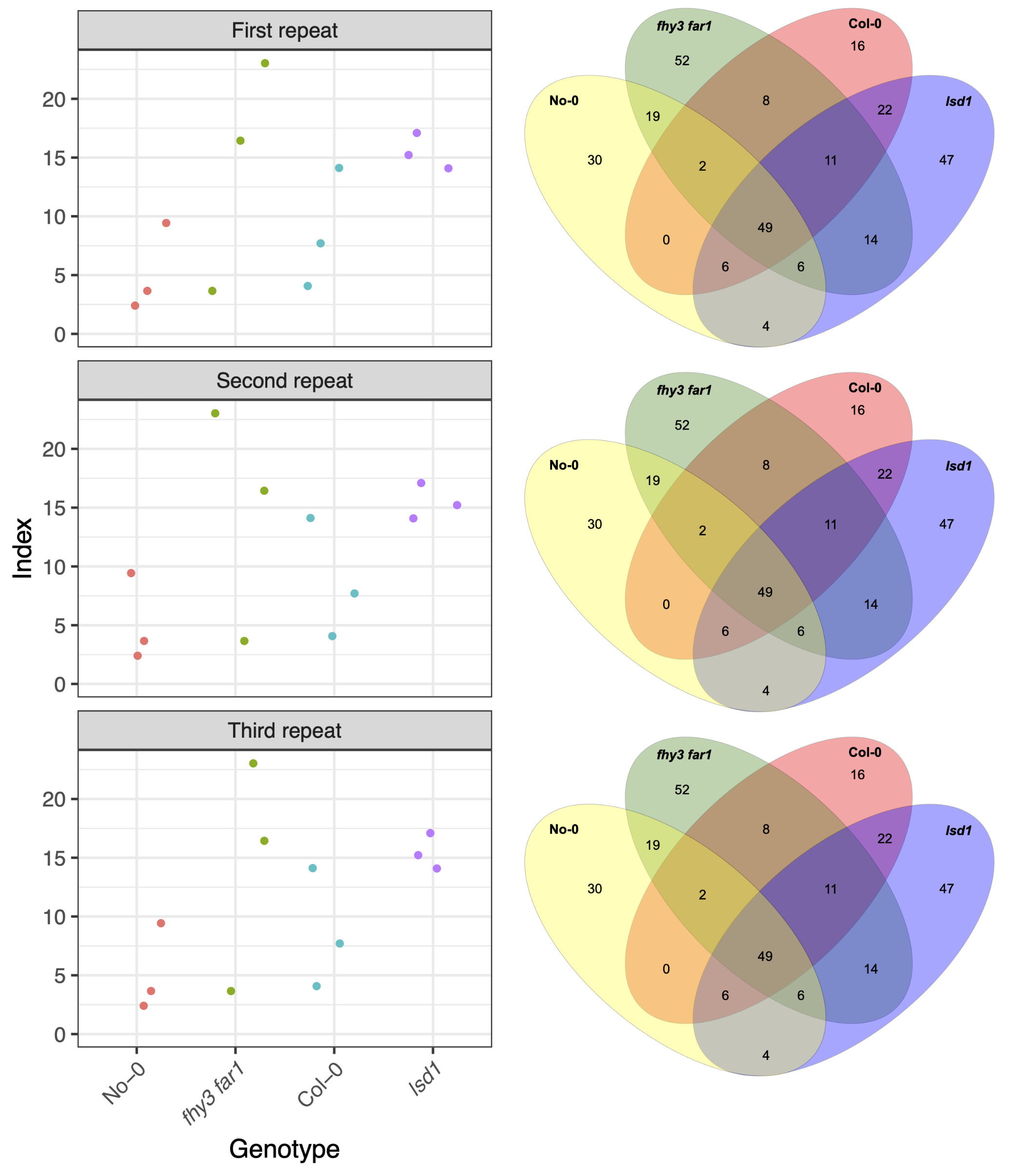
**

Fig. S2. Three repetitions of subsampling used for data analysis. Left panel: inverse Simpson diversity index values of bacterial phyllospheric communities. Right panel: Venn diagrams of phylotype presence and absence across four genotypes in each subsampling repeat. from two WT (No-0 and Col-0) and two mutant (*fhy3 far1* and *lsd1*) *A. thaliana* lines. Microbial DNA was isolated from aerial tissue 35 DAS in three biological replicates and subjected to NGS. Analysis carried out using mothur (v.1.44.3) (Schloss et al., 2009).

**
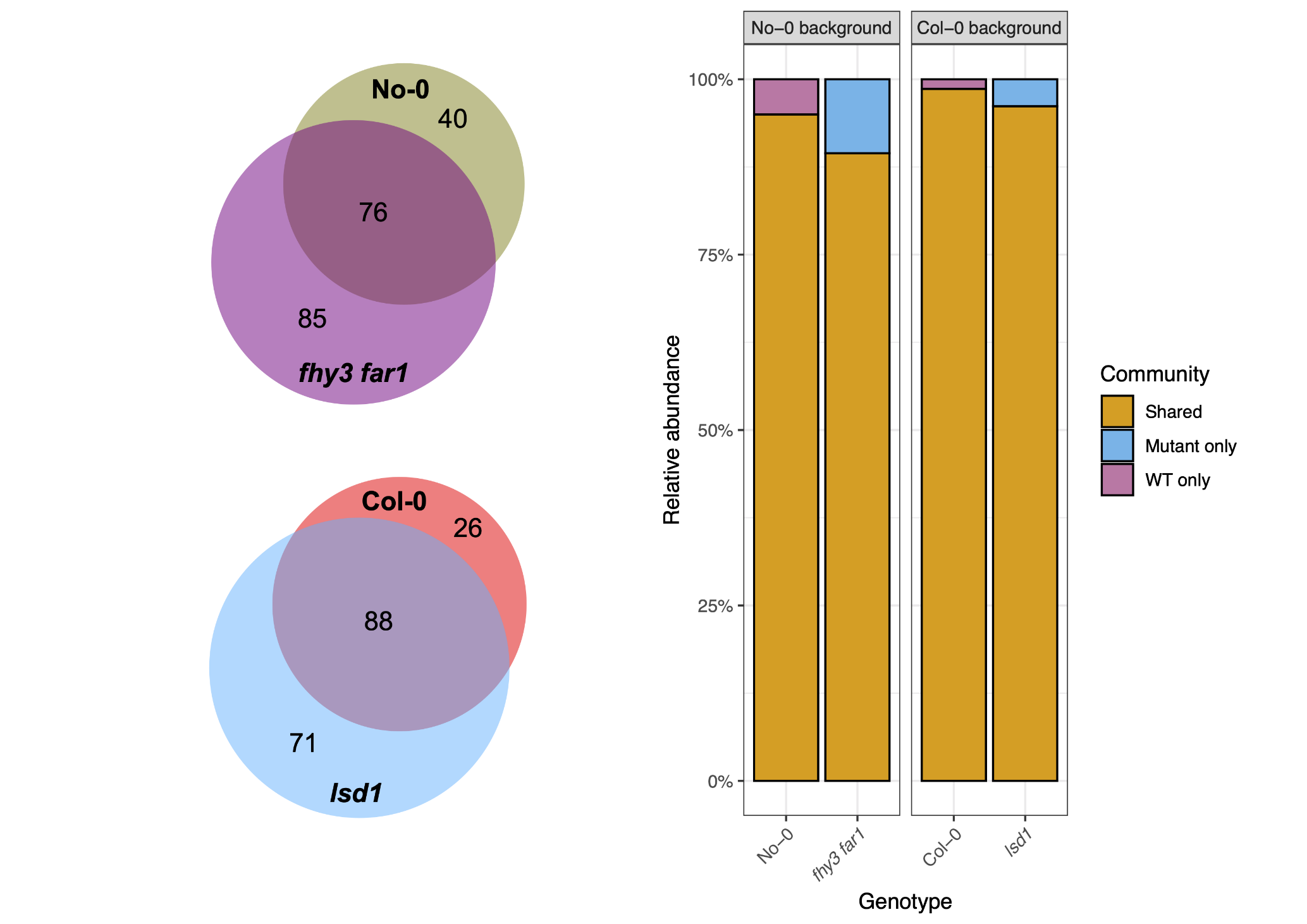
**

Fig. S3. Distribution of phylotypes within shared ecotype backgrounds. Left: Venn diagrams of phylotypes common to mutant and WT genotypes. Right: Relative abundances of phylotypes found in both genotypes of the same ecotype background (shared); and phylotypes found only in the mutant or WT genotype.

**References**

Schloss, P. D., Westcott, S. L., Ryabin, T., Hall, J. R., Hartmann, M., Hollister, E. B., et al. (2009). Introducing mothur: Open-source, platform-independent, community-supported software for describing and comparing microbial communities. *Appl. Environ. Microbiol.* 75, 7537–7541. doi:10.1128/AEM.01541-09.
